# Puerperal Eclampsia

**Published:** 1886-11-20

**Authors:** Walter A. Crow

**Affiliations:** Lecturer on Diagnosis in the Southern Medical College, Atlanta, Ga.


### Southern Medical Record.

A MONTHLY JOURNAL OF PRACTICAL MEDICINE.

☞All Communications must be addressed to R. C. WORD, M. D., Managing Editor.

"*Quicquid Præcipies Esto Brevis.*"

VOL. XVI. ATLANTA, GA., NOVEMBER 20, 1886. No. 11

### ORIGINAL AND SELECTED ARTICLES.

### PUERPERAL ECLAMPSIA.

BY WALTER A. CROW, M. D.,
Lecturer on Physical Diagnosis in the Southern Medical College, Atlanta, Ga.

[*Read before the Atlanta Society of Medicine.*]

*Puerperal Eclampsia* is a convulsion of epileptiform type, characterized by less of consciousness and of sensibility, together with tonic and clonic spasms, occurring during the puerperal state, and is, perhaps, received with more universal terror than any other accidents of the lying-in chamber. It is a scene once witnessed never to be forgotten.

*Etiology.*—This, so far as anything definite is concerned, is still an unsettled question. Rayere, in 1840, and Lever, in 1842,* and others, observed that the convulsions were associated with albumen in the urine. Frerchs supposed that the urea was converted into carbonate of ammonia, and as such exerted a deliterious influence ; but this theory seemed insufficient, since, of late, the examination of the urine of eclamptic patients shows quite a number in which there was no albumen present previous to the attack ; and for this reason Halbertsma† has modified that theory. He thinks that most cases of eclampsia are caused by the retention of the constituents of the urine, and this retention is not due to primary dis-

* Guy's Hospital Reports.

† Karl Schrœder, Manual of Midwifery.

ease of the kidneys, but to the pressure of the gravid uterus upon the uterus; that hyperæmia of the kidneys and diffused nephritis favor the occurrence of eclampsia. Traube's theory, as modified by Rosenstein, as to the uræmic symptoms, seems more satisfactory; i. e. a deficiency cf albumen in theblood and an increased pressure in the arterial system, which, though commonly produced by disease of the kidneys, may, under circumstances, arise independently of them. These two conditions, as essentials, are always present at least to a slight degree in parturient women; for a watery condition of the blood is peculiar to pregnant women, and during the pains the muscles of the body are in a state of activity, the pressure on the arterial system being very much increased. Now, if one or both of these conditions reach a very high degree, it may give rise to an eclamptic convulsion. The hydræma frequently becomes very intense in consequence of the loss of albumen through the kidneys; therefore, the greater predisposition in partruient women who suffer from Bright's disease.

Sir James Y. Simpson, in his work on Obstetrics, gives the following as supposed causes of convulsions:

1. Plethoric conditions of pregnancy.
2. Difficult labor.
3. State of atmosphere.
4. Dropsical diathesis.
5. Albuminuria.
6. Toxæmia—(*a*) blood charged with urea; (*b*) blood charged with carbonate of ammonia; (*c*) blood charged with kreatine and extractive matter; (*d*) blood charged with alkaloidal poisons.
7. Hydræmia, leading to œdema of the brain under increased blood pressure in the arteries.

The causes as given by Dr. Meadows* are: (1) Special irritability of the nervous system; (2) Irritation in some distant part of the body, especially the uterus or the digestive organs, accompanied by a morbid sensitiveness of the nerve centers; (3) Congestion of the brain or spinal cord, or both; (4) Sudden and violent mental emotion; (5) Morbid irritation of the uterus from the pressure of too much or too little blood. Dr.Taylor Smith observes, in application of the theory of reflex action promulgated by the late Marshall Hall, that "the true puerperal convulsion can only occur when the central organ of this system, the spinal marrow, has been acted on by an excited condition of an important class of its incident nerves, namely, those passing from the uterine organs to the

* **Meadows' Midwifery.**

spinal center—such excitement depending on pregnancy, labor or the puerperal state."

I will not discuss these different theories separately, but merely give two clinical facts apparently conflicting, that must not be lost sight of in studying the etiology of this disease :

First. It is not rare to see cases in which convulsions break out in pregnancy which were not preceded by albuminuria.

Second. Albuminuria, even with œdema, has been noted, and no convulsions has appeared.

We will next notice the morbid leision or *Pathology* of this disease, in order to better understand its supposed etiology, which we will also notice again. In studying the pathology of this, as well as other disease, we get the most satisfactory results by carefully observing the *post-mortem* changes. It is true that similar changes will not necessarily arise in the early steps of the disease, nor in those patients who recover ; still we can draw from this, facts which can be got by no other means, that will illustrate the phenomena observed in the living.

Braun, in describing the *post-mortem* appearance, gives the following : "The brain shows anæmia, œdema, diminished consistency. Hyperæmia of the membranes is rare, also intermeningeal apoplexy ; when it does occur, Kiwisch regards it as secondary—produced by impeded circulation of the blood ; œdema constant in lungs ; heart empty and flaccid, usually ; spleen large, which is generally the case during gestation and child-bed ; and the condition of the kidneys he divides into three different stages : 1. Hyperæmia ; 2. That of exudation and commencing metamorphosis ; and, 3, Atrophy or the stage of retrogression and dissolution of the glandular substance."

Dickenson thinks the morbid changes in the kidneys are such as obstructive or venous congestion would be apt to produce, and are allied to those changes which occur as the result of heart disease, with attendant venous repletion ; but probably of a more serious character when of uterine origin than of cardiac.

Dr. Mascorel, as reported by Depaul,* states that in the autopsies he made the kidneys were perfectly healthy or simply congested.

Now, from the facts before us, it seems to me that the most plausible conclusion can be reached only by taking from each of these different theories their salient points and adjusting them as a whole, which has been very cleverly done by Robert Barnes.

* Barnes' Obstetric Midwifery.

He gives as his theory: Several conditions concur to cause the associated disorder; these are (1) the "hydræmic state of gestation, leading to imperfect nutrition of the nervous centers, increasing (2) the normal nervous tension and irritability, and (3) the normal muscular tension. With these comes (4) blood-poisoning from imperfect elimination of waste stuff by the kidneys, and other emunctories"; or, as Fordyce Barker puts it, albuminuria, hydræmic anæmia, uræmia and primiparity. He also adds heredity and atmospheric influences.

*Prognosis.*—The prognosis, on the whole, is considered bad; still quite a goodly number of such patients recover; so our decision should be greatly modified by, especially, the condition of the kidneys and brain. Puerperal mania is not an uncommon sequella; meningeal hemorrhage is always to be feared when the convulsions continue for several hours. But there are occasional cases with the most unfavorable symptoms that make a good recovery; and to illustrate this I will give in detail a case that came under my observation:

Mrs. McC——, age thirty, was well advanced with her second pregnancy when her husband came to me and said his wife was suffering from swollen feet and legs, and he wanted me to send her some medicine. On inquiry, I found that the amount of urine passed was very little, and still a constant desire to urinate. As it was in the country, where physicians dispense the medicines, I sent her the following: ℞. Potas. cit. ℥i; syrup simp. ℥i; aquæ ℥vii; tinc. digitalis ʒiss. To take one tablespoonful every four hours; also to give a saline cathartic, and see that her bowels were kept open. I heard nothing more of the case until about 5 o'clock on the morning of the 25th of November, 1883, when I was called to attend her confinement (ten days from the time I sent the medicine). I found the patient, to my surprise, very much swollen (œdematus); not only her feet and legs, but body, arms and face; the skin was thickened and rough; but, on examination, I found that labor was progressing favorably. The os had dilated to the size of a silver dollar, the cervix relaxed and patulous; also a sufficient amount of secretions. The presentation was vertex L. O. A. She had had a good action on her bowels and emptied her bladder before I came; the pains were regular, and everything seemed to be progressing nicely. In about two hours (7 a. m.) I delivered her of twins (girls); the placenta was expelled in due time, and the uterus contracted down firmly. I gave no ergot, as the amount of blood lost was only moderate. I left her at half-past 8 feeling "all right," as she expressed it. At 1 p. m. I was summoned in great haste. On reaching her bedside, I found my patient in a very hard spasm ; her face was of a livid dusky hue, which made the scene really a frightful one. I dispatched a runner for a bottle of chloroform, and proceeded at once to open the median cephalic vein of the right arm ; took, I suppose, about 8 ounces of blood. This seemed to relax the spasm, and she passed into a relaxed condition, the breathing being of a sterturous snoring character. I at once used the catheter, but failed to get any urine. As soon as the patient was able to swallow I gave her potas. brom., grs. xx ; chlor. hydrate, grs. v, in a little water. At 2 p. m. I noticed the spasm returning, but kept off the severity of the convulsion by giving chloroform. She soon relapsed into the sterturous breathing, as before. I then placed *three* drops of croton oil on the root of her tongue; waited three-quarters of an hour, and then gave an enema of warm soapsuds, but failed to get an action from the bowels.

4 p. m.—Patient has had another spasm ; gave chloroform; used catheter, got about one tablespoonful of highly colored urine which contained an abundance of albumen, as shown by heat and nitric acid ; no action on bowels yet.

5 p. m.—Condition the same ; have to give chloroform on the slightest return of the spasm, as it seems to govern them to some extent. Dr. W. L. Dunn, who had been sent for, arrives. We gave potas. bromide, grs. xx ; chloral hydrate, grs. iv per bowel.

7 p. m.—Don't notice any material change ; she had a very hard spasm about forty minutes ago ; took from 12 to 14 ounces of blood from left arm ; no action on bowels ; no water passed.

12, midnight.—Patient has been kept under the influence of chloroform most of the time since 8 o'clock, on account of a return of the convulsions as soon as its influence passed off ; no action on the bowels yet ; no urine in bladder ; have given her an enema of castor oil, ℥ ss ; croton oil, gtts. iij ; soapsuds, warm, O i. It was retained half an hour, and then allowed to come away, but failed to cause any action on the bowels.

26th, 2 a. m.—Condition about the same ; got her to swallow a little bromide mixture, after which she seemed to revive a little ; opened her eyes and looked about for the first time, but soon began to show symptoms of a spasm.

4 a. m.—On account of a very hard spasm, we tried to get more blood, but failed. Have given grs. xx pulv. purgans; no urine yet; used dry cups over the kidneys.

6 a. m.—Have given no chloroform for an hour and a half ; she lies with her eyes partly shut ; snores very loud ; there seems to be some phlegm in her throat ; no action on bowels ; no urine. At this time I was compelled to be absent for three or four hours ; feeling confident my patient would be dead before I could get back, I asked Dr. D. to stay until 9 o'clock, if she lived untill then.

12 m.—To my surprise I find my patient still alive ; condition about the same as when I left. Dr. D. left at 10 o'clock, of course thinking the case hopeless. I learned from the friends that she had had no spasm during my absence. Have not tried to give her anything ; no action on bowels ; no urine passed. So here I was, as it seemed, my hands tied, and was almost prostrate from the fatigue and worry of twenty-four hours. Thought, perhaps I had pushed the means within my reach already too far. Surely *six drops* of croton oil was dangerous, and to give more would be hazardous ; but as a last resort in a hopeless case I put *three* drops of croton oil on the root of her tongue.

2 p. m.—Patient had a copious action from bowels, and I think passed some urine, but still lies motionless and unconscious.

3 p. m.—Continues to have copious watery discharges from bowels about every half hour ; used catheter and drew off 1 pint urine very highly colored and of ammoniacal odor ; forehead seems moist ; think she is breathing a little better.

6 p. m.—The discharges are wonderful ; she revives a little ; when spoken to sharply will open her eyes ; anæsthesia almost complete ; her tongue very much swoolen, caused by being lacerated by the teeth during the spasm. It is with difficulty that we can get her to swallow a little chicken broth and cream toddy.

27th, 6 a. m.—Not much change during the night ; continues to lie in one position ; snores loudly and occasionally moans ; took some nourishment ; bowels acted on twice ; kidneys acting well ; had to use catheter.

6 p. m.—Patient very pale ; skin moist ; the œdema considerably diminished ; begins to use her hands ; when aroused, she makes a muttering, incoherent noise ; can't understand anything she says, which I think is partly due to the condition of her tongue. Continue to give broths, rice-water and eggnog. Very good action on bowels in forenoon ; kidneys not so active ; temperature normal ; pulse 100, weak. Ordered cit. potass. and tincture digitalis every four hours during the night.

28th, 8 a. m.—Patient rested better last night ; does not snore so loud ; can use her arms a little, but seems restless at times. I gave grs. x pulv. purgaus at midnight ; kidneys acted very free during the night, but still obliged to use catheter ; bowels acted well at 4 a. m. ; continues to take nourishment.

Dec. 1.—For the last two days she has improved slowly ; bowels regular ; kidneys acting very well ; skin moist and flabby ; the œdema pretty much gone ; is gaining use of her limbs ; mind getting clearer ; can understand the most she says ; complains of her tongue. Continue the cit. potas. and digitalis mixture every six hours. The nurse reports the locial discharge about natural.

Dec. 4.—Continues to improve ; asked about the babies for the first time this morning ; but slight amount of albumen in urine to-day ; color bright ; bowels regular ; œdema entirely gone ; appetite good ; complains of pain in her side and stomach. Ordered sulph. quiniæ, grs. v ; sulph. morphia, gr. $\frac{1}{8}$, t. i. d.

Dec. 7.—General condition much better, but suffers from neuralgic pains ; used morphia hypodermically sometimes, and have applied cups along the spine. Put her on iron, nux vomica and quiniæ.

Dec. 10.—Patient resting easy ; seems to be doing very well, although very weak.

She continued to improve, and in five weeks was able to sit in a chair by the fire ; convalesence continued, and she made a good recovery.

### CASE II.

Mrs. H—— ; age, twenty-five ; primapara ; of nervous temperament ; was confined January, 1884 ; presentation L. O. A. Everything seemed favorable, except an insufficiency of the expulsory power of the pains after the head had passed the brim of the pelvis and was well down into the sacrum. I would certainly have used forceps, if they had been at hand ; but in about three hours she was delivered of a fine, large child ; the uterus contracted down nicely, the placenta coming away of itself in about twenty minutes. Just at this time I noticed, by the peculiar expression of my patient's face, that she was going to have a convulsion ; it was a very severe one, lasting some eight minutes. I at once opened a vein in her arm, bled her freely, and as soon as she could swallow gave brom. potas. grs. xx ; chloral-hydrate, grs. v, in water. She soon became quiet, and never had a return of the convulsions, and made a good recovery in the usual time.

I consider both of these cases of peculiar interest, as the cause of the convulsions are entirely different.

Case I, I think, was purely urenemic. I learned afterwards the lady had suffered from acute nephritis once or twice after being exposed to cold ; while Case II is a type of exaggerated nervous tension. caused by nervous reflex excitability. There was no albumen in her urine previous to the attack, and no œdema.

### TREATMENT.

This may be classed under three heads: 1. Prophylactic; 2. Remedial, and 3. Restorative.

(1) *Prophylactic Treatment.*—This, perhaps, has more immediate bearing in the majority of cases to the treatment of albuminuria. Dr. Tyler Smith says in regard to this: "It has been said that this disorder cannot be arrested during pregnancy; but I have never met with a case that resisted treatment, unless it had been neglected until the end of gestation." Fordyce Barker states that his experience will not warrant so strong an assertion; yet he rarely encounters puerperal convulsions when the previous detection of albumen has led him to be particularly apprehensive of their occurrence—by relieving the renal congestion with salines and hydragogue laxatives and mild diuretics, and improving the blood with iron and chlorate potassa, and the general tone of the system by a nutricious diet, excluding nitrogenized food; also proper hygenic regulations, we may hope to do a wonderful amount of good in preventing an attack. It is claimed by some that venesection may be used, under this head, in cases where the indications call for it, to relieve congestion. Dr. Barker also considers the use of chloroform and early delivery in suspected cases as a prophylactic measure, approving of a timely and judicious use of the forceps. The use of morphia, to relieve the cephalic pain, is still a mooted question. We have good authority both for and against it. Barker is inclined to favor its use. The hot water or vapor bath is of high repute in such cases. In the obstetrical department of the Vienna General Hospital it seems really to be considered as a "sheet-anchor," both as a prophylactic and a remedial agent. The manner of using the bath* is to place the patient in a bath-tub filled with water of a temperature above 99°, which is then covered with heavy blankets, leaving the face free, and the temperature of the water is gradually raised to 110° to 112° F. She remains in the bath about half an hour; cold cloths are put on the head to relieve the cephalic sensation. While in the bath, the patient drinks large quantities of water; on emerging from the bath, she is covered with a warm sheet and enveloped in an upper and lower layer of thick blankets, so that only the face is exposed. Within a few minutes free perspiration is observed, and the sweating is continued for two or three hours. These baths may be repeated once daily *proranata.* While resorting to this as a prophylactic measure—which I think is of considerable value—the physician should never forget the probability of bringing on premature labor.

* American System of Medicine, Vol. 4.

Under this heading, I also wish to notice the position of the patient as being of considerable importance. I refer to that class of patients who have to keep the recumbent position most of the time, and are already suffering from congested kidneys. These patients, instead of lieing flat on their backs, which most of them do, if they would lie upon their sides, changing, of course, from one to the other, they would not only relieve the congested kidney but remove the direct pressure of the gravid uterus from both the ureters and kidneys.

(2) *Remedial.*—Chloroform is, no doubt, entitled to the first place under this heading. Dr. Barker thinks that this agent alone has done more than all other resources known to our art, in diminishing the fatality from puerperal convulsions—putting the estimate of lives saved at 50 per cent. Venesection, in my opinion, is entitled to the second place. This practice has, no doubt, been greatly abused and, for this reason, abandoned to some extent; still, the taking from 14 to 16 ounces of blood at the beginning of an attack, from those patients that will bear bleeding, will generally stop the convulsions. Repeated bleeding or bleeding late in an attack is condemned by our best authorities. Chloral hydrate is held in high repute by a great many able writers; the maximum dose should not exceed grs. xx. I prefer combining it with bromide potas., say, grs. xx bromide to grs. x of chloral hydrate. Eleterium, croton oil, and pulvis purgans are the most efficient hydrogogues; of these, I prefer croton oil for two reasons: 1st. It is not so liable to be a spurious article, and, 2dly, you can give it to the patient by placing it on the root of the tongue, when you can't give eleterium. Diuretics of these, the citrate or the acetate of potassa, and digitalis are the best. Robert Barnes considers nitrite of amyle as desirable to relax the muscular spasm. Nitro-glycerine is claimed to be cxtremely promising by some. Pilocarpin has been tried, but the experiments are not encouraging. If the birth has not taken place, the child should be delivered at once, not hastily, but using every precaution not to injure the mother.

(3) *Restorative Treatment.*—This occupies our attention when labor is over, the convulsions cease, and the albumen decreasing, in trying to restore tone to the different organs whose functions have been impaired. The proper treatment, then, consists in rest, sedatives, light diet—the latter of which should form a large element, avoiding stimulants. Iron is indicated, the tinct. ferri chlor. being the best, and should be followed by salines; keep the skin in good condition by baths, and maintain good hygenic surroundings. By these means we may hope to do a great deal of good in restoring health to the unfortunate afflicted mother.

